# Condom use behaviour among people living with HIV: a seven-country community-based participatory research in the Asia-Pacific region

**DOI:** 10.1136/sextrans-2017-053263

**Published:** 2017-11-08

**Authors:** Keshab Deuba, Verena Kohlbrenner, Sushil Koirala, Anna Mia Ekström

**Affiliations:** 1 Department of Public Health Sciences, Karolinska Institutet, Stockholm, Sweden; 2 Programme to Foster Innovation, Learning and Evidence in HIV and Health Programmes of German Development Cooperation, GIZ, Bonn, Germany; 3 Asia Pacific Network of People Living with HIV/AIDS, Bangkok, Thailand; 4 Department of Infectious Diseases, Huddinge, Karolinska University Hospital, Stockholm, Sweden

**Keywords:** HIV infections/prevention and control, risk/taking

## Abstract

**Objectives:**

We examined the prevalence of inconsistent condom use and its correlates among people living with HIV (PLHIV) in the Asia-Pacific region.

**Methods:**

Between 1 October 2012 and 31 May 2013, a total of 7843 PLHIV aged 18–50 years were recruited using targeted and venue-based sampling in Bangladesh, Indonesia, Lao People’s Democratic Republic (PDR), Nepal, Pakistan, Philippines and Vietnam. Logistic regression was used to explore the association between condom use behaviour and demographics, social support, stigma and discrimination and various health-related variables.

**Results:**

Overall, 43% of 3827 PLHIV practised inconsistent condom use at sexual intercourse with their regular partner. An even higher proportion, 46% of 2044 PLHIV admitted that they practised unprotected sex with a casual partner. Participants from Lao PDR reported the lowest prevalence of inconsistent condom use for both regular and casual partners, while participants from the Philippines had the highest risk behaviour. Inconsistent condom use was significantly associated with belonging to a key population (drug user, sex worker or refugee subpopulation), not knowing that condoms are still needed if both partners are HIV positive, having a regular partner whose HIV status was either positive or unknown, having experienced physical assault and not receiving antiretroviral treatment.

**Conclusions:**

This large seven-country study highlights a high prevalence of inconsistent condom use among PLHIV in the Asia-Pacific region. In addition to knowledge-imparting interventions, the adoption and expansion of the ‘Test and Treat’ strategy could help to maximise the prevention benefits of antiretroviral treatment.

## Introduction

Despite a historic decline in new HIV infections and AIDS-related deaths over the past decades,^1^ the global target to reduce sexual and injection-related HIV transmission among youth and adults by 50% from 2010 to 2015 was not achieved because new adult HIV infections remained stagnant.^2^ The expectation that treatment as prevention^3–5^ dramatically would reduce the number of new HIV infections has only partially been fulfilled. Based on the findings from 61 studies, inconsistent condom use of people living with HIV (PLHIV) was related to having as a HIV-positive sexual partner, having less knowledge about HIV and believing that condom use decreases pleasure.^6^ There has also been a debate whether receiving antiretroviral therapy (ART) is linked to inconsistent condom use given fairly widespread awareness of treatment as prevention, and a review of 25 studies found no increase in inconsistent condom use in relation to receiving ART.^7^ However, the same review^7^ includes only one study conducted among PLHIV from the regions most affected by HIV: East and Southern Africa, West and Central Africa and the Asia-Pacific. Although the number of new HIV infections declined by 31% overall in the Asia-Pacific region since the year 2000, the success in preventing new HIV infections has differed significantly at the national level. In Nepal, new HIV infections declined by 79%, while the number is increased by fourfold in Indonesia.^8^

This study analysed a very large population-based cohort with data from seven countries in the Asia-Pacific region (Bangladesh, Indonesia, Lao People’s Democratic Republic (PDR), Nepal, Pakistan, Philippines and Vietnam) collected through community-based participatory research (CBPR) so-called CAT-S (community access to HIV treatment care and support services study). In 2014, the number of new HIV infections in the seven countries amounted to around 114 000, of which 61% occurred in Indonesia, 18% in Pakistan and 13% in Vietnam.^8^

CAT-S is a collaborative effort between researchers, community members and organisational representatives from the Asia-Pacific Network of PLHIV community members based in Bangladesh, Indonesia, Lao PDR, Nepal, Pakistan, Philippines and Vietnam who actively participated in different phases of the research to assess the prevalence of inconsistent condom use and associated risk factors in these seven countries, all experiencing concentrated HIV epidemics. This is defined as having an HIV prevalence of over 5% in subpopulations, such as people who inject drugs, sex workers, men who have sex with men (MSM) or refugees while having an HIV prevalence of less than 1% in the general population.

## Methods

We obtained quantitative data from the baseline data collection of the CAT-S. CAT-S involved 59 data collection sites across Bangladesh, Indonesia, Lao PDR, Nepal, Pakistan, Philippines and Vietnam and was designed to monitor and document factors related to access to HIV treatment and care and support services for PLHIV. Between 1 October 2012 and 31 May 2013, a total of 7843 PLHIV were recruited using modified targeted snowball sampling and facility-based sampling (online supplementary figure).^8^ To be eligible for the study, participants had to be between 18 and 50 years of age, had to self-report being diagnosed with HIV at least 3 months before the date of the interview and had to provide written informed consent. The study process, setting, sample size, study tools and sampling technique have been described in detail previously.^9^

10.1136/sextrans-2017-053263.supp1 Supplementary file 1

### Measures

A semistructured questionnaire was used to collect data through face-to-face interviews with a broad range of variables. Only variables relevant to the research question are reported in detail.

#### Outcome variable

Condom use behaviour in the past 6 months was assessed both at sexual intercourse with a regular partner and at sex with a casual partner (‘In the past 6 months, how frequently did you use condoms when you had sex?’). A regular partner was defined as a spouse or partner in a relationship with the interviewee for at least 3 months, and a casual partner was defined as any other sexual partner. Condom use behaviour was dichotomised into consistent use (‘always’) and inconsistent use (‘never’, ‘sometimes’ or ‘most of the time’).

#### Independent variables

Age, sex, education, occupation, income, living area, non-governmental organisation or community-based organisation membership and belonging to key populations (MSM, transgender, drug users, refugee, migrant workers and sex workers) at high risk of becoming infected with HIV were recorded as demographic characteristics. Factors having an established or theoretical association (eg, social support^10^) with condom use behaviour were taken into consideration (see online supplementary appendix 1).

10.1136/sextrans-2017-053263.supp2 Supplementary file 2

### Statistical analysis

Mean, SD and range were computed for continuous variables and frequencies for categorical variables. Logistic regression was used to examine the association between the independent variables and the outcome. Svy: logistic of STATA was used to fit the statistical model for complex survey data (in our case, 7 countries and 59 sites). All independent variables presenting a p value of ≤0.20 in the simple logistic regression and the control variable were included in the multiple logistic regressions. Two multiple logistic regression models with condom use behaviour as the outcome were computed, one among participants with a regular partner and one among participants with a casual partner. The variance inflation factors (VIFs) indicated no problematic multi-collinearity (all VIF values ≤1.51). All statistical tests were based on a significance level of a p value ≤0.05, and CIs were set at 95% confidence level.

### Ethical statement

Participants were informed about the objectives and procedure of the study, and only those who were willing to give written informed consent or a thumbprint were enrolled. Furthermore, participants were made aware of their rights as participants and confidentiality was assured. Also, a code of conduct on confidentiality, questionnaire storage and analysis was developed and used. Participants were given small financial incentives for participation: US$5 in Lao PDR, Indonesia, Nepal and Vietnam; US$10 in Bangladesh, Pakistan and the Philippines.

## Results

In total, 7843 PLHIV were recruited across the seven countries. Selected participant characteristics are displayed in online supplementary table. Of the 7843 PLHIV recruited, 53.9% had a regular partner. Among those, 55.0% indicated their regular partner’s HIV status as positive, 34.7% as negative and 10.3% as unknown.

10.1136/sextrans-2017-053263.supp3 Supplementary file 3

**Figure 1:**
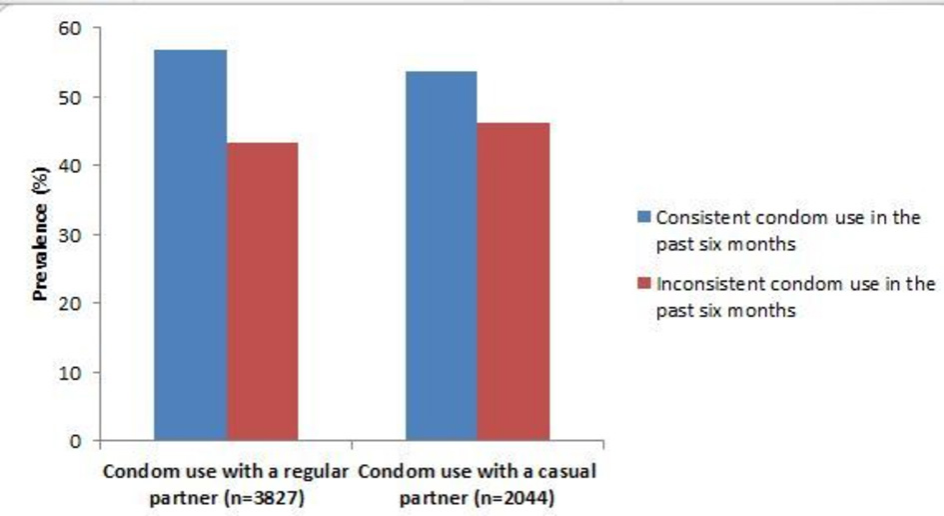
Condom use behaviour with a regular or a casual sexual partner among people living with HIV from seven countries of the Asia-Pacific region.

In the past 6 months, 3827 (48.8%) participants had sexual intercourse with a regular partner, of which 43.3% reported inconsistent condom use. Furthermore, 2044 participants (26.1%) had had sex with a casual partner in the past 6 months, of which 46.2% reported inconsistent condom use. Figure 1 shows the prevalence of inconsistent condom use for the two groups.

At the national level, participants from Lao PDR reported the lowest prevalence of inconsistent condom use for both regular and casual partners, while participants from the Philippines had the highest risk behaviour. Detailed data on the prevalence of inconsistent condom use at the national level are displayed in table 1.

**Table 1:** Prevalence of inconsistent condom use with a regular or a casual sexual partner among people living with HIV in Bangladesh, Indonesia, Lao PDR, Nepal, Pakistan, Philippines and Vietnam

Country	Prevalence of inconsistent condom use n(%)	
	Regular partner	Casual partner
Bangladesh	22.5 (88)	48.6 (17)
Indonesia	45.3 (320)	41.4 (237)
Lao PDR	13.6 (37)	16.7 (12)
Nepal	41.8 (317)	31.1 (71)
Pakistan	32.3 (98)	36.8 (63)
Philippines	62.6 (218)	59.6 (320)
Vietnam	55.2 (577)	52.5 (225)
Total	43.3 (1655)	46.2 (945)

### Condom use behaviour with regular and casual partners

Inconsistent condom use was reported by 43.3% of the 3827 participants who had sexual intercourse with their regular partner in the past 6 months. Results of the multiple logistic regression assessing the association between inconsistent condom use and the independent variables are displayed in table 2.

**Table 2:** Multiple logistic regression assessing the association between inconsistent condom use with regular and casual partners among people living with HIV

Characteristic	Inconsistent condom use with regular partner (n=1 655)		Inconsistent condom use with casual partner (n=945)	
	n (%†)	AOR‡ (95% CI)	n (%†)	AOR§ (95% CI)
Sex				
Male	891 (38.2)	1	651 (45.2)	1
Female	747 (51.3)	1.55 (0.91 to 2.62)	240 (53.3)	1.71 (1.08 to 2.70)*
Transgender	17 (44.7)	1.15 (0.25 to 5.34)	54 (35.1)	0.83 (0.49 to 1.38)
Living area				
Large town or city	954 (48.7)	1	676 (48.5)	1
Small town	442 (37.1)	0.69 (0.53 to 0.93)*	160 (41.0)	0.73 (0.44 to 1.23)
Rural area	258 (38.5)	0.60 (0.42 to 0.86)*	109 (42.3)	0.69 (0.43 to 1.11)
Key populations¶				
Sex worker	249 (60.1)	1.57 (1.219 to 2.05)**		
Refugee	100 (62.9)	2.74 (1.709 to 4.44)**	73 (76.0)	5.15 (2.09 to 12.66)**
Prisoner	64 (57.7)	1.61 (0.75 to 3.45)		
Physical assault				
No			851 (45.6)	1
Yes			94 (53.7)	57 (1.25 to 1.99)**
Illicit drug use				
Never			607 (44.8)	1
Past user			213 (43.7)	1.26 (0.77 to 2.07)
Current user			125 (62.2)	1.90 (1.08 to 3.36)*
Place of HIV diagnosis				
Private hospital	234 (45.4)	1		
Government hospital or VCT centre in a hospital	945 (44.1)	0.73 (0.44 to 1.22)		
VCT centre in an NGO	345 (37.5)	0.63 (0.51 to 90.77)**		
Others^††^	131 (53.25)	0.92 (0.44 to 1.90)		
Current enrolment in ART				
No			376 (53.0)	1.40 (1.09 to 1.81)*
Yes			569 (42.7)	1
HIV-related literacy^‡‡^				
Condoms are still needed when the viral load is undetectable	1136 (40.9)	0.93 (0.65 to 1.33)		
Sexual risk behaviour can lead to a drug resistance type of HIV	1180 (41.4)	0.91 (0.67 to 1.21)	676 (44.5)	0.83 (0.59 to 1.18)
Condoms are still needed if both partners are HIV positive	1193 (40.0)	0.52 (0.46 to 0.62)***	737 (44.8)	0.76 (0.66 to 0.89)***
HIV status of the regular partner				
Negative	342 (25.9)	1		
Positive	1 086 (50.6)	3.03 (1.92 to 4.80)**		
Unknown	227 (63.6)	2.76 (1.69 to 4.51)**		
Condom availability				
Always or mostly	1 172 (35.9)	1	631 (36.8)	1
Sometimes	315 (88.0)	12.60 (7.76 to 20.49)***	231 (95.9)	46.30 (20.51 to 104.51)***
Never	159 (86.9)	8.52 (5.24 to 13.8)***	82 (93.2)	25.71 (9.11 to 72.48)***

*p≤0.05; **p<0.01; ***p<0.001.

†Percentages are based on row frequencies.

‡Adjusted for age, occupation, non-governmental organisation/community-based organisation membership, income, social support from friends, housing instability, self-rated health, drug and alcohol consumption, children and the desire to have children, time since HIV diagnosis, current enrolment in ART, disclosure of the own HIV status to the partner, sex with someone else and the variables listed within the table.

§Adjusted for age, education, non-governmental organisation/community-based organisation membership, social support from family, self-rated health, time since HIV diagnosis, place of HIV diagnosis and the variables listed in the table.

¶Multiple responses were possible. Not belonging to the respective key population functioned as reference category in the regression. All key populations were included in bivariate analysis, but only those categories that were significant or marginally significant (>0.05 but <0.10) in bivariate analysis were presented in table.

††Participants were asked to specify; most frequent answers were ‘abroad’ and ‘mobile VCT’.

‡‡Not knowing the respective fact functioned as reference category.

AOR, adjusted OR; ART, antiretroviral therapy; VCT, voluntary counselling and testing.

Inconsistent condom use was reported by 46.2% of the 2044 participants who had sexual intercourse with a casual partner in the past 6 months. Results of the multiple logistic regression assessing the association between inconsistent condom use and the independent variables are displayed in table 2.

Inconsistent condom use with both regular and casual partners was associated with being female, being a refugee, lack of condom availability and lack of awareness that condoms are still needed if both partners are HIV positive (table 2). PLHIV living in a small town or a rural area were less likely to report inconsistent condom use with a regular partner. Other factors that increased the likelihood of inconsistent condom use with a regular partner were being sex workers and getting diagnosed with HIV at a private hospital. PLHIV who indicated their partner’s HIV infection status as positive and participants who did not know their partner’s HIV infection were around three times more likely to report inconsistent condom use, compared with participants who indicated their partner’s HIV infection status as negative (table 2). PLHIV who had experienced physical assault and were current users of illicit drugs and who were not enrolled on ART were more likely to report inconsistent condom use with a casual partner.

## Discussion

Our findings suggest that the prevalence of inconsistent condom use with different sexual partners—regular or casual partners of unknown or different HIV status—is high (43%–46%) among PLHIV in this seven-country study in the Asia-Pacific region. Furthermore, women (when compared with men) and key populations (sex workers, refugees and drug users) were more likely to report inconsistent condom use. Other characteristics found to be associated with inconsistent condom use were living area (urban), poor HIV treatment literacy, place of HIV diagnosis (private hospital), regular partner HIV status (HIV positive and unknown status) and the experience of physical violence. Inconsistent condom use among PLHIV who were not receiving ART at the time of the interview or who did not know about ART (overall 24% of the study participants, but with great variation between countries from 3% in Lao PDR to 33% versus34% in Nepal and Philippines) can be seen as a missed opportunity to use treatment as prevention benefits of ART. Inconsistent condom use among PLHIV not only poses a threat for secondary transmission of HIV but also leads to transmission of drug-resistant HIV strains, which will ultimately hamper the effectiveness of ART.^11^

Across the study countries, participants from Lao PDR reported the lowest prevalence of inconsistent condom use for both regular and casual partners, whereas participants from Vietnam and the Philippines had the highest sexual risk behaviours. This finding is also consistent with previous research showing that inconsistent condom use is high among key populations (sex workers, drug users and MSM) living with HIV in Vietnam and Philippines, which is contributing rapidly to new HIV infection in these countries.^12 13^ In addition, the growing inhumane approach to address drug issues in the Philippines, affecting scale up of HIV prevention and treatment efforts,^14^ will definitely escalate risky behaviours of key populations in the future, especially among drug users in the Philippines.

In our study, women were more likely to report inconsistent condom use with different partners. Other studies indicate that poverty and entrenched gender inequities influence unprotected sexual behaviours among women through various pathways such as by increasing survival sex, low self-esteem and poor condom negotiation skills.^15–17^ In consistency with previous research,^18–20^ we found that PLHIV involved in sex work, drug users and refugees are more likely to report inconsistent condom use. A recent review that includes most of the studies from Asia found that sex workers often lack access to condoms due to their unsafe sex work environment (changes to the venue, management, policing policies and access to prevention), which influences their condom use behaviours.^21^ It is of major concern that National HIV Strategic Plans in most Asian countries so far have not prioritised refugees or internally displaced persons as a target population for HIV-related efforts,^22–24^ and it should be an urgent priority to implement programmes to address non-use of condoms and to improve condom accessibility among refugees/internally displaced persons living in both camp and non-camp setting in the Asia-Pacific region.

The unique feature of the epidemic in the Asia-pacific region is that the HIV infection remains concentrated among key populations and in the urban areas.w1 Our study found that PLHIV living in an urban area were more likely to report inconsistent condom use with regular partners. We also found that having experienced physical assault or having a regular partner of HIV-positive and unknown status is associated with inconsistent condom use, which is similar to results in other studies conducted among PLHIV in Brazil and six countries in Central America.w2 w3 PLHIV in our study with poor HIV treatment literacy (not being aware that condoms are needed if both sexual partners are HIV positive) were more likely to report inconsistent condom use with both regular and casual partners. Similarly, a study conducted among adult PLHIV in Ethiopia found that PLHIV with knowledge of HIV treatment were more likely to report consistent condom use.w4 This suggests that PLHIV in the Asia-Pacific region are practising unsafe sexual behaviour either because they lack knowledge of different aspects of HIV transmission or that they, regardless of this, still practice risky sexual behaviours. The earlier issue can be addressed by proper delivery of information during counselling process, but to address the latter reason, we have to conduct qualitative studies for an in-depth understanding of reasons behind the intentional practice of unprotected sex among PLHIV.

One of the encouraging findings of our study is that those PLHIV who were enrolled in ART are less likely to practice inconsistent condom use than PLHIV who were not enrolled in ART. Similarly, a systematic review of studies from low-income countries and other independent studies conducted in South Africa and six countries in Central America found a lower likelihood of unprotected sex associated with ART.w3 w5 w6 This also suggests that the implementation of the ‘Test and Treat’ strategy—most of the countries in the Asia-Pacific region started recommending treatment irrespective of CD4 count—will increase the ‘treatment as prevention’ benefits of ART.

CBPR is widely used in others fields to generate evidence,w7 but only to a limited extent in the area of HIV research. However, the current CBPR study suggests that this is a useful method to generateevidence for  response activities among PLHIV. The existing national network of PLHIV in each study country greatly facilitated the coordination and management of research activities including field implementation. The informal sharing of challenges faced by PLHIV on a daily basis to access ART also helped us to refine our study variables. Their involvement in the data collection also helped us to recruit hidden and marginalised PLHIV who were experiencing the dual burden of stigma due to their HIV status and belonging to one of the key populations (MSM, transgender and sex workers). However, the biggest challenges we faced is that the use of study findings for evidence-based action is not uniform in all study countries. The capacity and interest of PLHIV leaders or activists to use study findings differ between study countries.

## Conclusions

Our study findings provide evidence of high prevalence of non-use of condoms with regular and casual partners among PLHIV in Bangladesh, Indonesia, Lao PDR, Nepal, Pakistan, Philippines and Vietnam. In addition to knowledge-imparting interventions, the adoption and expansion of ‘Test and Treat’ strategy will help to reduce secondary transmission of HIV in the Asia-Pacific region. Behavioural change intervention should also target key populations such as PLHIV involved in sex work, drug users and refugees.

**Key messages**

- Inconsistent condom use with different partners was widely prevalent among people living with HIV (PLHIV) in the study countries.
- PLHIV from Lao reported the lowest prevalence of inconsistent condom use, while participants from the Philippines had the highest risk behaviour.
- Inconsistent condom use was associated with poor HIV treatment literacy, physical assault and not receiving antiretroviral therapy.
