# Supplementary figures and images for: Condom use behaviour among people living with HIV: a seven-country community-based participatory research in the Asia-Pacific region
Source: Sex Transm Infect. 2017 Nov 8;94(3):200–5. doi: 10.1136/sextrans-2017-053263 (PMC5969330; doi:10.1136/sextrans-2017-053263)

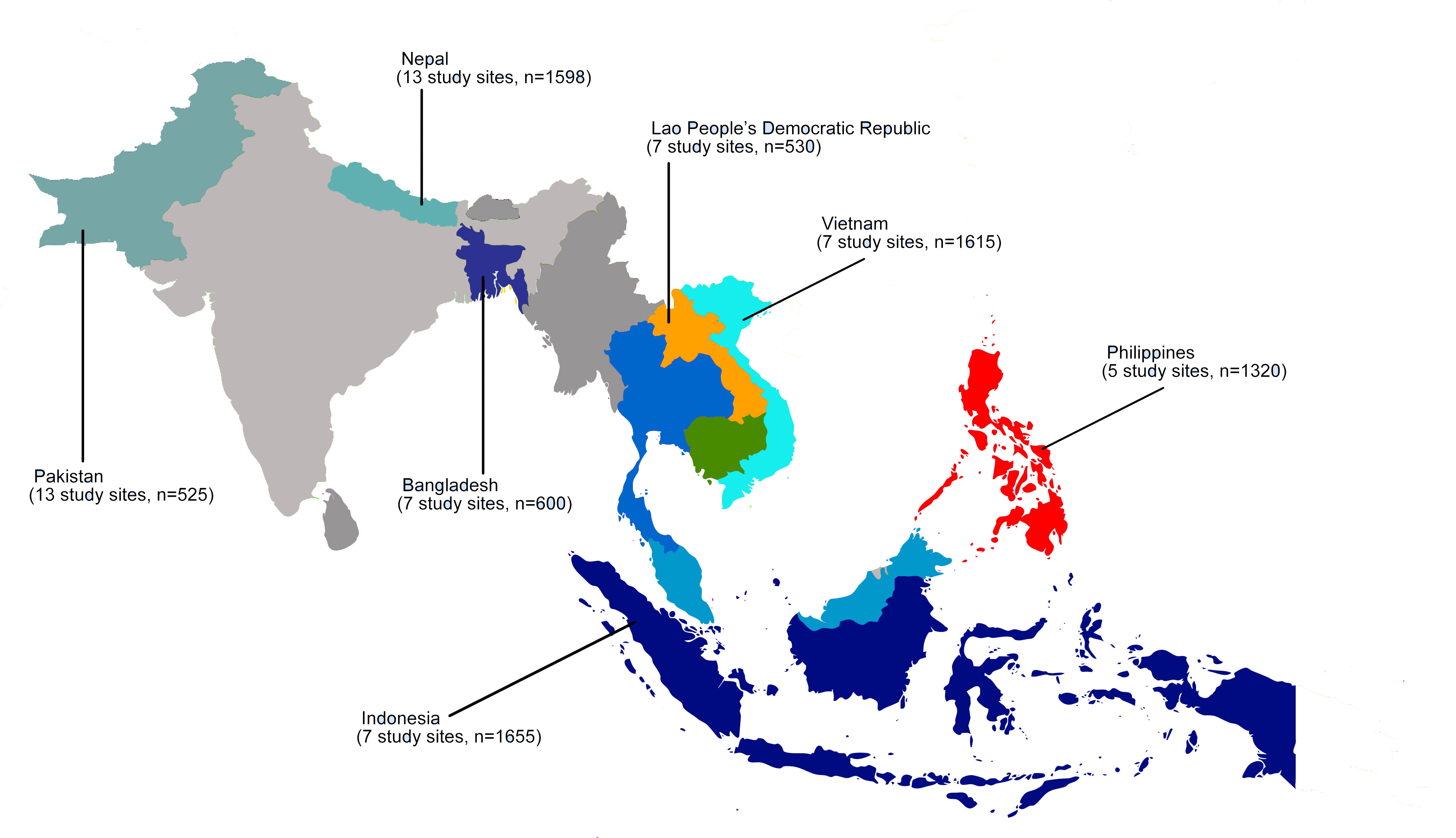

Supplement: Supplementary file 1 [file sextrans-2017-053263supp001.jpg]
