# Supplementary material for: Condom use behaviour among people living with HIV: a seven-country community-based participatory research in the Asia-Pacific region
Source: Sex Transm Infect. 2017 Nov 8;94(3):200–5. doi: 10.1136/sextrans-2017-053263 (PMC5969330; doi:10.1136/sextrans-2017-053263)
Supplement: Supplementary file 2 [file sextrans-2017-053263supp002.docx]

**Web Appendix 1. Independent variables**

Factors having an established or theoretical association with condom use behaviour were taken into consideration: social support, stigma and discrimination, general and reproductive health, HIV diagnosis, and HIV treatment and related literacy. To assess social support from family, friends, and significant others, the Multidimensional Scale of Perceived Social Support was used. Participants rated twelve statements (e.g. “I can talk about my problems with my family.”) on a scale ranging from 1 (“strongly disagree”) to 5 (“strongly agree”), and a social support score was calculated.

Measures on stigma and discrimination included physical assault and housing instability in the past twelve months, amongst others. To assess general and reproductive health, self-rated health, illicit drug use (“Have you ever used any illicit drugs?”; the response was categorized as “never used drugs” vs. “current drug user” vs. “former drug user”), and alcohol consumption (categorized as “never drinkers” vs. “former drinkers” vs. “current drinkers”) were recorded. Furthermore, participants were asked if they had children and/or if they had a desire to have children in the future. Time since the HIV diagnosis in years and place of the HIV diagnosis were recorded as measures related to the HIV diagnosis. Measures on HIV treatment included current enrollment in ART and related literacy, which was assessed asking participants to rate HIV-related statements (e.g. “Condoms are not needed if both sexual partners are HIV-positive.”) as either true or false. Participants who had sex with a regular partner in the past six months were further asked to indicate their partner’s HIV status, whether they had ever disclosed their HIV status to their partner, and whether they had sexual intercourse with someone other than their regular partner in the past six months. Condom availability (“Can you get a condom when you need it?”) was recorded and categorised into “always or mostly”, “sometimes”, and “never”.
