# Supplementary material for: Condom use behaviour among people living with HIV: a seven-country community-based participatory research in the Asia-Pacific region
Source: Sex Transm Infect. 2017 Nov 8;94(3):200–5. doi: 10.1136/sextrans-2017-053263 (PMC5969330; doi:10.1136/sextrans-2017-053263)
Supplement: Supplementary file 3 [file sextrans-2017-053263supp003.htm]

|  |  |  |
| --- | --- | --- |
| **Web-only table 1.** Selected demographic characteristics of PLHIV with a regular or a casual sexual partner. | | |
| **Characteristic** | **Frequency (%)** | |
|  | **Regular partner (n = 3 827)** | **Casual partner (n = 2 044)** |
| **Country** |  |  |
| Bangladesh | 391 (10.2) | 35 (1.7) |
| Indonesia | 707 (18.5) | 572 (28.0) |
| Lao PDR | 273 (7.1) | 72 (3.5) |
| Nepal | 759 (19.8) | 228 (11.2) |
| Pakistan | 303 (7.9) | 171 (8.3) |
| Philippines | 348 (9.1) | 537 (26.3) |
| Vietnam | 1 046 (27.3) | 429 (21.0) |
| **Age** |  |  |
| Mean (SD) | 34.7 (6.6) | 32.0 (6.8) |
| **Sex** |  |  |
| Male | 2 332 (60.9) | 1 440 (70.5) |
| Female | 1 457 (38.1) | 450 (22.0) |
| Transgender | 38 (1.0) | 154 (7.5) |
| **Education** |  |  |
| Illiterate | 385 (10.1) | 111 (5.4) |
| Can read and write/Primary level | 1 006 (26.3) | 324 (15.9) |
| Secondary level and above | 2 410 (63.0) | 1 592 (77.9) |
| Othera | 26 (0.7) | 17 (0.8) |
| **Occupation** |  |  |
| Unemployed | 657 (17.2) | 404 (19.8) |
| Employed | 3 170 (82.8) | 1 640 (80.2) |
| **Income (USD)** |  |  |
| Mean (SD) | 127.0 (169.2) | 188.7 (215.9) |
| **Living area** |  |  |
| Large town or city | 1 961 (51.3) | 1 394 (68.3) |
| Small town | 670 (17.5) | 390 (19.1) |
| Rural area | 1 190 (31.1) | 258 (12.6) |
| **NGO/CBO member** | 1 717 (44.9) | 706 (34.6) |
| **Key populationsb** |  |  |
| MSM | 390 (10.2) | 746 (36.5) |
| Lesbian | 10 (0.3) | 10 (0.5) |
| Sex worker | 414 (10.9) | 538 (26.4) |
| Injecting drug user | 1 091 (28.6) | 516 (25.3) |
| Refugee | 159 (4.2) | 96 (4.7) |
| Domestic migrant worker | 203 (5.3) | 42 (2.1) |
| International migrant worker | 628 (16.5) | 136 (6.7) |
| Prisoner | 111 (2.9) | 52 (2.6) |
| **Note.** NGO = nongovernmental organization; CBO = community-based organization; MSM = men who have sex with men; SD= Standard deviation  a Participants were asked to specify, most frequent answers were “vocational” and “can sign only”.  b Multiple responses were possible. | | |
